# Prediction of post-traumatic growth in the face of the COVID-19 crisis based on resilience, post-traumatic stress and social participation: A longitudinal study

**DOI:** 10.3389/fpsyg.2022.985879

**Published:** 2022-08-11

**Authors:** Paula Collazo-Castiñeira, Rocío Rodríguez-Rey, Helena Garrido-Hernansaiz, Silvia Collado

**Affiliations:** ^1^Department of Psychology, Faculty of Human and Social Sciences, Universidad Pontificia Comillas, Madrid, Spain; ^2^Department of Geriatrics, Hospital Universitario Ramón y Cajal (IRYCIS), Madrid, Spain; ^3^Department of Education and Psychology, Centro Universitario Cardenal Cisneros, Alcalá de Henares, Spain; ^4^Department of Psychology and Sociology, Faculty of Social and Human Sciences, University of Zaragoza, Teruel, Spain

**Keywords:** COVID 19 pandemic, lockdown 2020, post-traumatic growth, post-traumatic stress, resilience, social participation

## Abstract

The COVID-19 crisis has generated a severe and negative psychological impact worldwide. Despite this, it is also possible to experience post-traumatic growth (PTG). This study aimed to longitudinally explore the prevalence of PTG in the Spanish population and test a predictive model for PTG from resilience, post-traumatic stress symptoms (PTSS), and participation in social activities. Data were collected longitudinally in March, July, and November 2020 *via* an online survey. About 20% of the sample showed moderate-high levels of PTG, with no significant differences over time. The predictive model explained 19% of the variance in PTG, showing that the inverse relation between resilience and PTG was mediated by PTSS. Additionally, participation in social activities acted as a predictor of PTG. Women, young people, those who had lost their job and people who had experienced COVID-19 symptoms or the loss of a loved one presented higher PTG. Thus, people have experienced positive changes (PTG), but these did not protect them from adverse symptomatology (PTSS).

## Introduction

The pandemic situation generated by COVID-19 has had a serious impact on the population’s mental health worldwide (Da Silva Neto et al., 2021; Prati and Mancini, 2021; Mahmud et al., 2022). In Spain, in March 2020, between 15 and 41% of the population presented moderate-severe post-traumatic stress symptoms (PTSS; González-Sanguino et al., 2020; Rodríguez-Rey et al., 2020). Despite the adverse impact generated by this crisis, the experience of post-traumatic growth (PTG) was also reported (e.g., Yeung et al., 2022; Zhang et al., 2022), understood as the perception of positive psychological changes after going through a potentially traumatic situation (Tedeschi and Calhoun, 2004). Thus, Tedeschi and Calhoun (2004) proposed that people have a series of beliefs that can be challenged by a traumatic experience, allowing their subsequent reconstruction and leading to the development of PTG. This construct has gained presence in the literature on traumatic experiences in recent years (Helgeson et al., 2006).

PTG has been studied as a result of different types of traumatic experiences, for example, individual adverse situations like living with HIV or suffering a traffic accident (Nishi et al., 2010; Garrido-Hernansaiz et al., 2017), but also collective traumatic experiences like natural disasters such as earthquakes or nuclear accidents (Pérez-Sales et al., 2005; Kaye-Kauderer et al., 2019). Furthermore, PTG has also been reported following past health crises, such as the SARS epidemic in 2003 (Cheng et al., 2006). All of the mentioned adverse situations generate feelings of anxiety, fear, and worry in the population (Cheng, 2004; Esterwood and Saeed, 2020) due to their unpredictability uncertainty, and the risks involved. However, in the case of the COVID-19 health crisis, its impact goes beyond health implications, as the measures put in place to prevent its propagation have significant repercussions on the population’s everyday life (e.g., lockdowns, quarantines, curfews, social distancing, etc.). Even though these measures have affected everyone, people show different trajectories following these adversities, from the resilient ones who neither presented significant levels of perceived stress nor PTG, to the “resurgent” ones who experienced both perceived stress and PTG (Baños et al., 2022). Given that the COVID-19 health crisis has affected the population globally and differently, it becomes a relevant context for exploring in which cases PTG flourishes, how it evolves, and which variables (both personal and contextual) are important in its development.

The construct of PTG has received some criticism, such as, for example, that it is not a real experience of positive changes, but a passing and illusory response that perhaps acts as a coping strategy (Kaur et al., 2017). However, the presence of PTG over time (i.e., longitudinally) has not been systematically studied (see, as an exception, Cheng et al., 2020; Zhao et al., 2021). This makes it difficult to clarify whether PTG is temporary or, on the contrary, persists over time.

The development of PTG has been studied in the context of the COVID-19 health crisis. These studies, mostly from a cross-sectional approach, reported a significant prevalence of PTG in populations of different nationalities (Ikizer et al., 2021; Kalaitzaki, 2021; Matos et al., 2021), including Spain (Prieto-Ursúa and Jódar, 2020; Vázquez et al., 2021). The sociodemographic profile associated with higher levels of PTG as a result of the COVID-19 health crisis was consistent with that found in previous crises (Helgeson et al., 2006; Vishnevsky et al., 2010), with women and younger people experiencing PTG to a greater extent. Additionally, in these studies on PTG during COVID-19, different contextual variables were found to be related to the levels of PTG reported. In particular, it seemed that experiencing harsher and more adverse conditions was related to higher levels of PTG, which was also consistent with previous studies (Laufer and Solomon, 2010). For example, having experienced changes in the workplace as a result of the pandemic (e.g., having lost their employment, suffered a salary reduction; Ikizer et al., 2021; Na et al., 2021) and having had greater contact with the disease (i.e., having suffered the disease or having presented symptoms) showed a positive relationship with PTG (Prieto-Ursúa and Jódar, 2020; Zhang et al., 2022), as well as having suffered the loss of a loved one due to COVID-19 (Prieto-Ursúa and Jódar, 2020; Chen and Tang, 2021).

Presenting a higher level of perceived concerns or risks derived from the COVID-19 crisis also had a positive relationship with PTG (Hyun et al., 2021; Ikizer et al., 2021; Na et al., 2021; Yeung et al., 2022). For example, perceived risk of unfavorable economic changes (Ikizer et al., 2021) or concern for physical and mental health in the wake of this crisis (Na et al., 2021) was associated with higher PTG. In this sense, the most frequent concerns in the early stages of the pandemic, which in turn were related to more PTSS, were in reference to the economic situation, a loved one contracting COVID-19, and not knowing when this crisis would end (Rodríguez-Rey et al., 2020). Despite these findings, in the context of COVID-19, there are limited longitudinal studies that evaluate the importance of sociodemographic and contextual variables on PTG in the long term. Therefore, the first objective of this study was to evaluate the temporal stability of PTG in the context of the COVID-19 health crisis and explore the possible influence that sociodemographic and contextual variables had on the development of PTG over time.

In addition, there are other, less-studied variables in this context that could be relevant to the development of PTG, such as participating in leisure activities after an adverse experience (Chun and Lee, 2010). Thus, Chen et al. (2020), in their meta-analysis, found that sport acts as a facilitator in the development of PTG. Another factor that could be related to the development of PTG is participation in collective activities and rituals, which in past crises were related to a reconstruction of positive beliefs based on support and social cohesion (Páez et al., 2013). However, in the context of COVID-19, this has been frustrated by the limited physical contact due to preventive measures such as home confinement (Ammar et al., 2020). Even so, an increase in the sense of belonging and social and family cohesion was reported after this confinement (Saiz et al., 2021; Waters et al., 2021), similarly to other previous community catastrophes (Somasundaram, 2004; Pérez-Sales et al., 2005). This, at least in Spain, could be due to the collective activities carried out remotely: collective applause for health workers at 8 p.m., events *via* social networks, or community cooperation activities (e.g., doing the shopping for those who could not). To our knowledge, the possible influence of having participated in these activities during the earliest phases of the pandemic has not been previously studied. Given the key role that social connection and support play in the development of PTG (Prati and Pietrantoni, 2009; Rzeszutek and Gruszczyńska, 2018), it is relevant to examine the effect of participation in these activities during the earliest phases of the pandemic in the development of PTG. In fact, in the context of COVID-19, social support has been a significant predictor of PTG. For example, Matos et al. (2021) found that social connection predicted higher PTG consistently across all countries assessed. In turn, Mo et al. (2021) reported that, in frontline nurses, social support was one of the main predictors of PTG. Taking into account the positive effect that this connection seems to have on the development of PTG, the second objective of this study was to evaluate the effect of social participation as a possible facilitator of PTG in the context of COVID-19.

There are two variables, so far unmentioned, that are key in the study of traumatic experiences. First, PTSS are a relevant indicator of the degree of affectation that an event has generated in an individual. Second, resilience, understood as the ability to adapt and recover more easily after experiencing adverse circumstances (Smith et al., 2008), is the main protective factor against experiencing PTSS (Levine et al., 2009; Bonanno et al., 2011). The relationship between these two variables and PTG is complex and controversial in the literature (Sawyer et al., 2010; Shakespeare-Finch and Lurie-Beck, 2014; Garrido-Hernansaiz et al., 2017; Rzeszutek and Gruszczyńska, 2018). Beginning with PTSS, and according to the theoretical paradigm of PTG, PTG is the result of reconstructing basic personal beliefs that had been previously challenged by an adverse event (Tedeschi and Calhoun, 2004). For such a challenge to personal beliefs to occur, the event must be sufficiently striking (Helgeson et al., 2006). Therefore, it is normal that when a person suffers more intense PTSS, there may also be a higher PTG (Rodríguez-Rey et al., 2020). However, the literature reports direct, inverse, and non-existent relationships between PTSS and PTG (Shakespeare-Finch and Lurie-Beck, 2014). Given this, various meta-analyses have found that these two variables coexist, supporting both: a linear relationship between the two and a curvilinear one. However, they have particularly supported the latter, where moderate PTSS levels are those related to higher PTG levels (Shakespeare-Finch and Lurie-Beck, 2014; Tsai et al., 2015). Therefore, higher growth does not necessarily imply a lesser experience of PTSS (Tedeschi and Calhoun, 2004), as apparently derived from some studies that found an inverse relationship. In addition, studies have shown that the predictive value of PTSS for PTG is maintained longitudinally (Zhou et al., 2015). Considering the above, it is necessary to use PTSS as a predictor of the development of PTG and, in fact, it has been used in the latest predictive models of PTG in the context of COVID-19 (e.g., Lau et al., 2021; Northfield and Johnston, 2021).

Continuing with resilience, and considering the premise that to develop PTG it is necessary to experience PTSS at least to some extent, resilience (as a protective factor that predicts lower levels of PTSS) would then have to be inversely related to PTG. In addition, such a relationship would have to be mediated by PTSS. Actually, this is what the theoretical model proposes: those with greater resilience will be less affected by the traumatic event, challenging their beliefs to a lesser extent and, consequently, limiting the potential reconstruction of these (Westphal and Bonanno, 2007; Tedeschi and McNally, 2011). Nevertheless, the results of previous studies mostly indicated a positive relationship between resilience and PTG (Dong et al., 2017; Rzeszutek and Gruszczyńska, 2018), with limited studies to support the opposite (Garrido-Hernansaiz et al., 2017; Rodríguez-Rey and Alonso-Tapia, 2019). Thus, the third and final objective pursued by this study was to test a predictive model based on the theoretical paradigm of PTG (considering the mediating role of PTSS in the relationship between resilience and PTG) and contribute to the resolution of the controversies in the literature in this regard. Additionally, this predictive model will also verify the facilitating role that participating in collective activities might play in the development of PTG, not just considering the necessary condition of an adverse situation, but also the positive aspects that could facilitate the development of PTG in the aftermath of trauma.

**FIGURE 1:**
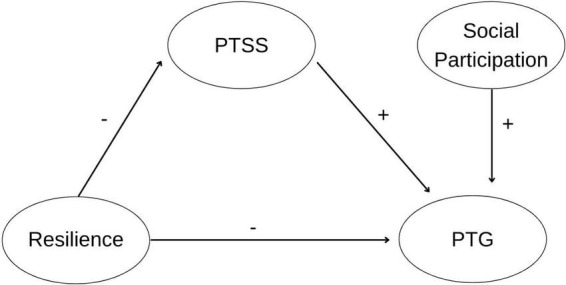
Hypothetical relations between variables in the PTG predictive model.

To synthesize, there is evidence of the emergence of PTG in the face of the COVID-19 crisis, however, the existence and etiology of PTG are unclear. Thus, this study has three aims: (1) to longitudinally explore the evolution of PTG generated as a result of the COVID-19 crisis and evaluate its temporal stability, as well as identify contextual and sociodemographic variables associated with its development; (2) to evaluate the effect of social participation as a possible facilitator of PTG in the COVID-19 crisis; and (3) to delve deeper into the study of PTG development by testing the predictive model of PTG development based on the theoretical postulates previously laid out (see Figure 1). Specifically, we expect to find (a) an inverse relationship between resilience and PTG, (b) a mediation effect of that relationship *via* PTSS, and (c) a significant positive effect of social participation on PTG.

## Materials and methods

### Participants

The participants were adults living in Spain during the COVID-19 pandemic. In March 2020 (T1), 3055 people completed the questionnaire (75.1% women, *M* = 32.15 years). In July 2020 (T2), 855 people participated again, and in November 2020 (T3), 592 people filled out the questionnaires for a third time. The sociodemographic characteristics of the T3 sample are reflected in Table 1.

**TABLE 1:** Association between demographic variables and PTG in T3 (*N* = 592).

Variables	*N* (%)	PTG			
		*M* (*SD*)	*t*/*F*	*p*	*g*/η^2^
**Gender^2^**			5.00^1^	<0.001	0.46
Female	468 (79.32)	15.13 (9.76)			
Male	122 (20.68)	10.72 (8.38)			
Other	2 (0.30)	9.5 (13.44)			
**Country of origin**			–1.41	0.17	–0.31
Spain	560 (94.59)	14.04 (9.52)			
Other	32 (5.41)	17 (11.63)			
Region			1.69	0.09	0.14
Madrid	325 (54.9)	14.81 (9.70)			
Other	267 (45.1)	13.46 (9.56)			
**Marital status**			3.26	0.01	0.02
Married/ cohabiting with a partner	266 (44266 (44.93)	12.7 (9.54)^a^			
In a relationship but not cohabiting	137 (23.14)	15.64 (9.74)^b^			
Widow(er)	3 (.51)	9.67 (6.11)^ab^			
Separated/ divorced	19 (3.21)	15.05 (10.41)^ab^			
Single	167 (28.21)	15.4 (9.46)^b^			
**N° of children**			1.44	0.23	0.01
None	410 (69.26)	14.63 (9.48)			
One	66 (11.15)	14.48 (9.91)			
Two	91 (15.37)	12.46 (10.13)			
Three or more	25 (4.22)	12.84 (9.80)			
**Education level**			0.82	0.56	0.01
Primary education	3 (0.51)	19.33 (13.2)			
Compulsory secondary education	8 (1.35)	12.88 (11.99)			
Post-compulsory secondary education	44 (7.43)	12.16 (9.38)			
Professional training	69 (11.66)	14.09 (9.50)			
University degree	282 (47.64)	14.43 (9.55)			
Master’s degree	137 (23.14)	14.91 (9.77)			
Ph.D.	49 (8.28)	12.8 (9.95)			
**Monthly income**			1.28	0.26	0.01
<1000 €	48 (8.11)	15.42 (10.19)			
1000 – 1500 €	90 (15.20)	14.41 (10.36)			
1500 – 2000 €	94 (15.88)	15.99 (9.05)			
2000 – 2500 €	102 (17.23)	13.66 (9.79)			
2500 – 3000 €	73 (12.33)	14.3 (10.19)			
3000 – 3500 €	66 (11.15)	12.88 (8.49)			
>3500 €	114 (19.26)	12.91 (9.19)			
**Age groups^3^**			3.34	0.006	0.03
18 – 24	160 (27.03)	15.57 (9.30)^a^			
25 – 34	172 (29.05)	15.11 (9.81)^a^			
35 – 44	106 (17.91)	11.25 (8.94) ^b^			
45 – 54	100 (16.89)	13.64 (10.18)^ab^			
55 – 64	41 (6.93)	14.98 (9.11)^ab^			
65-77	13 (2.20)	11.38 (10.69) ^ab^			

Categories with a different superscript letter show statistically significant differences between them for the PTG variable.

^1^Homoscedasticity could not be assumed for these variables, thus *t*-test results adjusted for non-homogeneous variances were used; in the case of ANOVA, *post hoc* Games–Howell tests were used.

^2^Given the low number that responded “other,” only men and women were included.

^3^*M* = 35.02, *SD* = 12.9.

### Instruments

#### Instruments used at T1 (March 2020)

##### Sociodemographic data

Participants provided their age, gender, country of birth, region, marital status, number of children, level of education, and monthly income per family unit.

##### Impact of event scale-revised

The Impact of Event Scale-Revised (IES-R; Weiss and Marmar, 1996; Weiss, 2007), validated in Spain (Báguena et al., 2001), is a self-report questionnaire of 22 items that measure the PTSS of the last 7 days before the experience of a traumatic event. It has three subscales: Avoidance (eight items), Intrusion (seven items), and Hyperactivation (seven items). The answer format consisted of a scale ranging from 0 (*not at all*) to 4 (*extremely*). In the present study, the tool was adapted such that this event referred to the COVID-19 crisis (e.g., “*Any reminders brought back feelings about COVID-19 health crisis”*). In this study, an adequate internal consistency (α = 0.94) was obtained for the scores of the total scale.

##### Brief resilience scale

The brief resilience scale (BRS; Smith et al., 2008) is a six-item self-report questionnaire that measures resilience as the capacity to recover from an adverse event (e.g., “*I tend to bounce back quickly after hard times*”). The answer format consisted of a scale ranging from 1 (*strongly disagree*) to 5 (*strongly agree*). It is unifactorial and a higher score indicates greater resilience. We used the Spanish version validated by Rodríguez-Rey et al. (2016), whose scores had good internal consistency in the present study (α = 0.81).

##### Situation in the workplace

Participants provided information on their employment status at the time, whether they had undergone significant changes in employment status due to the pandemic, whether they perceived a risk of losing their job for this reason and whether their income had decreased.

##### Contact with COVID-19

Participants indicated whether they had had symptoms characteristic of this disease or if they had been tested for COVID-19.

##### Concerns

Participants reported the degree of concern regarding various situations arising from the health crisis (e.g., I am concerned about my psychological state during this crisis). The response format was from 1 (*no or hardly*) to 4 (*very*).

##### Leisure activities during confinement

Participants indicated what leisure activities they carried out during this period (e.g., practicing sports, watching series, etc.).

#### Instruments used at T2 (July 2020)

##### Post-traumatic growth inventory-short form

The Post-traumatic growth inventory-short form (PTGI-SF; Tedeschi and Calhoun, 1996; Cann et al., 2010) is a 10-item self-report derived from Tedeschi and Calhoun’s original 21-item version (1996) that evaluates PTG (e.g., “*I have a greater appreciation for the value of my own life*”). Garrido-Hernansaiz et al. (2022) carried out the validation of the instrument in a Spanish sample in the context of the COVID-19 pandemic, obtaining a final 8-item instrument with four subscales and two items per subscale: Appreciation for life and new opportunities, Relationship with others, Personal strength, and Spiritual change. It has a Likert response format ranging from 0 (*I did not experience this change as a result of my crisis*) to 5 (*I experienced this change to a very great degree as a result of my crisis*). The PTG score was computed as the mean of the item scores. A score of three or more is indicative of a PTG of at least mid-grade (Tedeschi and Calhoun, 1996; Rodríguez-Rey and Alonso-Tapia, 2017). The internal consistency of the scores was adequate in the two evaluations (T2 and T3) of this study, for both the total scale (α = 0.87 – 0.88) and the subscales (Appreciation for life: α = 0.80 – 0.83; Relationship with others: α = 0.70 – 0.69; Personal strength: α = 0.83 – 0.84; Spiritual change: α = 0.68 – 0.70).

##### Personal losses

Participants indicated whether they knew anyone who died from COVID-19.

##### Social participation during the general confinement

Participants indicated their participation in collective activities during home confinement, including three categories: (1) activities of recognition and gratitude toward health workers (e.g., applause at 8 p.m.); (2) community aid and collaboration activities (e.g., doing the shopping for someone in need); and (3) activities *via* social networks (e.g., concerts).

#### Instruments used at T3 (November 2020)

Participants filled out the PTGI-SF again and reported their employment status, contact with the disease, concerns, and personal losses.

##### Leisure hours

Participants indicated how many hours a day, on average during the previous week, they spent on leisure activities away from home or meeting with non-cohabitants (0 = *Less than 1 h*; 4 = *More than 5 h*).

### Procedure

The study protocol was approved by the ethics committee of the University that led the study and was not pre-registered elsewhere. Between March 17th and 24th 2020 (T1), participants were contacted by social networks (Facebook, Twitter, Instagram, WhatsApp, and LinkedIn) requesting both their participation and the dissemination of the questionnaire, following the snowball method. After providing their informed consent, the participants went on to complete the questionnaire. At the end of the questionnaire, they were asked for permission to contact them at a later time, providing an email address or a telephone number. In July 2020 (T2), the 1598 participants who gave their contact details in T1 were contacted again, and in November 2020 (T3), the 855 who filled out the questionnaire in T2 were contacted. The data from T1 and T2 was used in previous reports (Rodríguez-Rey et al., 2020; Garrido-Hernansaiz et al., 2022). The present study reports data from T3 participants, using some variables measured at T1 and T2 to study their associations with T3 variables (i.e., the long-term effects of the COVID-19 crisis).

### Statistical analysis

First, we verified whether the sample loss between T1 and T2 and between T2 and T3 was due to random factors or, conversely, the participants who continued to participate differed significantly from those who only completed the questionnaire in T1 or T2. To this end, Student’s *t*-tests were performed for continuous variables (e.g., PTSS) and Chi-square tests for categorical variables, such as sex.

Next, to assess potential method bias in the scales used for this study, Harman’s one-factor test was performed. If one factor accounts for most of the measures’ covariance (usually interpreted as more than 50%), it would indicate that method bias is present (Podsakoff et al., 2003). In this study, a variance of 29.25% was obtained, indicating that the relations among these variables are not due to method bias.

We then calculated the percentage of participants who presented PTG of at least mid- or high-degree. To study the evolution of PTG, measured in T2 and T3, a paired-samples *t*-test was used. To study to what extent the variables evaluated in T1 and T2 related to the levels of PTG in T3, different statistical tests were performed. Thus, to explore the relationship between PTG and dichotomous variables (e.g., sex), Student’s independent samples *t*-tests were performed. For variables with multiple categories, one-factor ANOVAs were performed, using the *post hoc* Tukey analysis when the variances were homogeneous and the Games–Howell one when they were not. Additionally, the size effect was evaluated with Hedges’ *g* for Student’s *t*-tests (interpretation: negligible < 0.20 < small < 0.50 < medium < 0.80 < large) and η^2^ for ANOVA (interpretation: negligible < 0.01 < small < 0.06 < medium < 0.14 < large). An ancillary analysis was carried out, introducing relevant sociodemographic variables (*via* dummy variables) related to PTG in a multiple linear regression analysis. Also, bivariate correlation analyses were performed to explore the relationship between PTG and continuous (Pearson’s *r*) or ordinal (Spearman’s ρ) variables and quadratic and linear models were calculated to check whether the relationship between PTSS (T1) and PTG (T3) followed an inverted U shape.

To test the proposed theoretical predictive model, structural equation modeling (SEM) was employed using maximum likelihood as the estimation method. To assess the model fit, a mixed approach was used as recommended by Hu and Bentler (1999), including the absolute fit index χ^2^/df, two baseline close-fit indices (SRMR and RMSEA), and two incremental close-fit indices (CFI and TLI). The values indicative of good fit were ratio χ^2^/*df* < 3 (Hair, 2014), SRMR ≤ 0.08, RMSEA ≤ 0.06; CFI and TLI ≥ 0.95 (Hu and Bentler, 1999). To test the mediation effects, indirect effects were calculated using 10,000 samples from the bootstrap method, stipulating a 95% confidence interval. Statistical analyses were performed using AMOS Graphics 24.0 for SEM and SPSS 25.0. for the rest. All analyses were two-tailed and used a 95% confidence interval.

## Results

### Sample homogeneity (T1-T2-T3)

Statistically significant differences were found between those who continued participating in T2 and T3 and those who ended their participation in T1 or T2. On the one hand, those who participated in T2 were more likely to be women and older than those who abandoned the study after T1, with no other significant differences for the remaining variables. On the other hand, those who participated in T3 were older and with lower PTG and PTSS at T2 than those who dropped out after T2 (for more information see Supplementary Table 1).

### Descriptive statistics for resilience, post-traumatic stress symptoms and post-traumatic growth

The descriptive statistics for the study variables – Resilience, PTSS, and PTG – can be found in Table 2, reporting mean, standard deviation, minimum and maximum values obtained, as well as skewness and kurtosis. These values are reported for the entire sample and for males and females separately.

**TABLE 2:** Descriptive statistics for the study variables.

		*N*	*M* (*SD*)	Min	Max	Skewness (*SE*)	Kurtosis (*SE*)
PTSS (T1)	Total	592	27.16 (17.62)	0	84	0.62 (0.1)	–0.30 (0.2)
	Males	122	20.97 (16.45)	0	72	0.88 (0.22)	0.22 (0.44)
	Females	468	28.78 (17.60)	0	84	0.57 (0.11)	–0.36 (0.23)
Resilience (T1)	Total	592	19.87 (4.93)	6	30	–0.46 (0.1)	–0.14 (0.2)
	Males	122	20.98 (5.15)	6	30	–0.64 (0.22)	0.22 (0.44)
	Females	468	19.60 (4.81)	6	30	–0.42 (0.11)	–0.24 (0.23)
PTG (T3)	Total	592	14.20 (9.65)	0	40	0.36 (0.1)	–0.78 (0.2)
	Males	122	10.72 (8.38)	0	32	0.59 (0.22)	0.40 (0.44)
	Females	468	15.13 (9.75)	0	40	0.28 (0.11)	–0.86 (0.23)

### Post-traumatic growth levels and evolution

There were no significant differences in PTG levels between July (T2) and November (T3) 2020, *t*(591) = 1.39, *p* = 0.17. In T2, 22.2% of participants showed medium-elevated PTG levels – i.e., a score of 3 or higher (Tedeschi and Calhoun, 1996; Rodríguez-Rey and Alonso-Tapia, 2017) – (*M* = 15.15, *SD* = 9.51), while in T3, this proportion was 19.3% (*M* = 14.20, *SD* = 9.65).

### Relation between post-traumatic growth level and sociodemographic and contextual variables of COVID-19

#### Sociodemographic variables

Table 1 shows the descriptive data of the sociodemographic variables, as well as the relation of these with PTG in T3. We observed that women, younger participants, singles, and couples who were not cohabiting presented higher levels of PTG (i.e., those married showed significant lower levels of PTG than those single and those in a relationship but not cohabiting; regarding age, those aged 35–44 showed significantly lower levels of PTG than those aged 18–24 and 25–34). Effect sizes were small in all cases.

A multiple linear regression analysis was conducted, with PTG as criterion and the sociodemographic variables associated with PTG (i.e., gender, marital status, and age groups) as predictors. The results are included in Supplementary Table 2. Seven percent of the variance was explained [*R*^2^ = 0.07; *F*(9,582) = 4.71; *p* < 0.001]. The following variables emerged as relevant in the prediction of higher levels of PTG: female gender (as opposed to male gender), having a relationship but not living with the partner and being single (as opposed to being married or cohabiting with a partner), and an age of 25–34 or 55–64 (as opposed to an age of 35–44).

Regarding the participants’ employment situation, in March 2020 (T1), 90.6% of the sample reported undergoing changes in their work or studies. Between March and November (T2 and T3), 18.75% had to stop working or lost their job, while 65.2% had a salary reduction. Greater PTG was presented by the participants who lost their jobs between March (T1) and November 2020 (T3; *M* = 16.1, *SD* = 9.77), *t*(364) = –2.40, *p* = 0.02, *g* = –0.27, compared with those who did not (*M* = 13.44, *SD* = 9.74), and those who received a salary reduction (*M* = 15.11, *SD* = 9.73), *t*(590) = –3.17, *p* = 0.002, *g* = –0.27, compared with those who did not (*M* = 12.5, *SD* = 9.3).

#### Contact with COVID-19

Regarding the level of COVID-19 contact and its relationship with PTG, those participants who had greater contact with COVID-19 had higher levels of PTG. This occurred in those who suffered symptoms compatible with the disease (*M* = 15.77, *SD* = 9.64) compared with those who did not (*M* = 13.66, *SD* = 9.61), *t*(590) = –2.33, *p* = 0.02, *g* = –0.22; those who underwent diagnostic tests (*M* = 15.26, *SD* = 9.84) compared with those who did not (*M* = 13.01, *SD* = 9.33), *t*(590) = –2.81, *p* = 0.01, *g* = –0.23; and those who suffered the loss of a loved one (*M* = 15.06, *SD* = 9.86) compared with those who did not (*M* = 12.88, *SD* = 9.19), *t*(590) = –2.70, *p* = 0.01, *g* = –0.23.

#### Concerns

The level of concern of the participants regarding different issues throughout the three evaluations is shown in Table 3. Higher levels of concern were associated with higher PTG scores. Specifically, concern for one’s psychological state was the one, throughout all the evaluations, most related to PTG.

**TABLE 3:** Spearman correlation between different concerns (scored from 1 to 4) throughout the three collection times and PTG (*N* = 592).

	*M* (*SD*)	Spearman’s Rho	*p*
**Concerns in T1**			
Lack of capacity of the health system	2.94 (1.01)	0.03	0.44
COVID-19 infection of a loved one	3.3 (0.78)	0.07	0.07
Lack of food supply and medical devices (e.g., masks or gloves)	2.46 (0.99)	0.01	0.88
Insufficient measures by the government	2.83 (0.89)	0.08*	0.04
The economic impact of the pandemic	3.31 (0.74)	0.01	0.76
The situation of collective nervousness	2.92 (0.85)	0.04	0.35
Not knowing when this crisis will end	2.95 (0.89)	0.13**	0.001
My psychological state during this crisis	2.33 (0.99)	0.19***	<0.001
Mean level of concern	2.88 (0.54)	0.12**	0.006
**Concerns in T2**			
Getting infected by COVID-19	2.51 (0.84)	0.16***	<0.001
COVID-19 infection of a loved one	3.48 (0.69)	0.15***	<0.001
The economic impact of the pandemic	3.34 (0.70)	0.06	0.11
Not knowing when this crisis will end	3.13 (0.79)	0.13**	0.001
My psychological state during this crisis	2.27 (1.01)	0.24***	<0.001
The appearance of new outbreaks	3.25 (0.70)	0.16***	<0.001
Continue to use security measures	2.06 (0.92)	0.07	0.06
Others not maintaining security measures	3.36 (0.75)	0.09*	0.02
The impact COVID-19 is having on my life	2.52 (0.87)	0.20***	<0.001
Mean level of concern	2.88 (0.48)	0.24***	<0.001
**Concerns in T3**			
Getting infected by COVID-19	2.45 (0.88)	0.17***	<0.001
COVID-19 infection of a loved one	3.46 (0.74)	0.20***	<0.001
The economic impact of the COVID-19 pandemic	3.34 (0.74)	0.06	0.14
Not knowing when this crisis will end	3.15 (0.85)	0.17***	<0.001
My psychological state during this crisis	2.24 (1.02)	0.29***	<0.001
Continue to use COVID-19 safety measures	2.03 (0.96)	0.09*	0.02
Others not maintaining COVID-19 safety measures	3.21 (0.84)	0.09*	0.02
The impact COVID-19 is having on my life	2.46 (0.96)	0.20***	<0.001
COVID-19 vaccine availability	2.66 (1.13)	0.14***	<0.001
Whether the COVID-19 vaccine is safe or not	2.59 (0.89)	0.12**	0.003
Mean level of concern	2.76 (0.51)	0.27***	< 0.001

*p < 0.05, **p < 0.01, and ***p < 0.001.

#### Participation in social activities

Participation in social activities during the home confinement was positively related to PTG (see Table 4). Specifically, those who participated in the applause for the health workers at 8 p.m. and attended events on social media presented a higher PTG.

**TABLE 4:** Association between social participation during confinement (T2) and PTG in T3 (*N* = 592).

Variables	*N* (%)	PTG			
		*M* (*SD*)	*t*	*p*	*g*
Applause at 8 p. m.			–3.89	<0.001	–0.35
No	182 (30.74)	11.91 (8.94)			
Yes	410 (69.26)	15.22 (9.8)			
Community cooperation			–1.30	0.20	–0.11
No	297 (50.17)	13.69 (9.24)			
Yes	295 (49.83)	14.72 (10.04)			
Social media events			–2.80	0.005	-0.23
No	313 (52.87)	13.16 (9.39)			
Yes	279 (47.13)	15.37 (9.83)			
	***M* (*SD*)**	**Spearman’s ρ**	***p***		
Total participation	1.66 (0.96)	0.15	<0.001		

#### Leisure activities

As regards carrying out leisure activities, in March (T1), most participants (48% of participants from T3; *n* = 291) reported devoting less than an hour a day to leisure activities during the general home confinement. Carrying out various leisure activities was not related to PTG, except for physical exercise. In this sense, those who exercised (*M* = 15.31, *SD* = 9.48), *t*(590) = –2.95, *p* = 0.003, *g* = –0.24, showed higher PTG than those who did not (*M* = 12.99, *SD* = 9.72). In addition, a statistically significant and positive correlation was found between the number of leisure hours in July (T2) and the level of PTG in November (T3; ρ = 0.14, *p* = 0.001).

### Post-traumatic growth prediction: The role of resilience, post-traumatic stress symptoms, and social participation

Correlation analyses were performed to study the association between PTG at T3 and resilience and PTSS. A statistically significant and direct correlation was found between PTSS (T1) and PTG (T3; *r* = 0.30, *p* < 0.001), while resilience (T1) was inversely related to PTSS (T1; *r* = –0.34, *p* < 0.001) and PTG (T3; *r* = –0.08, *p* = 0.047). As regards a possible curvilinear relationship between PTG (T3) and PTSS (T1), the linear model had the same adjustment as the quadratic model (*R*^2^ = 0.09, *p* < 0.001 in both cases), being unable to establish the predominance of either.

**FIGURE 2:**
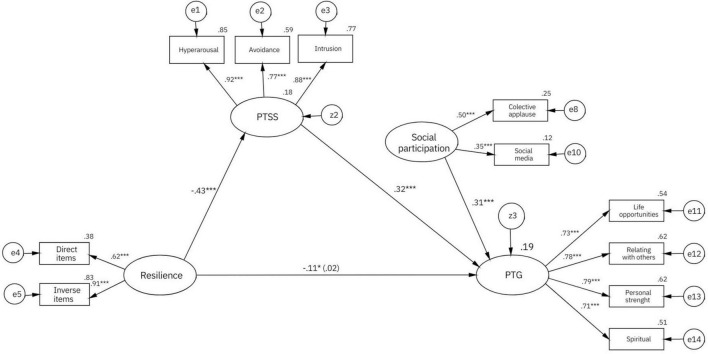
Predictive model, with standardized regression coefficients. The direct effect can be found between brackets (controlling for the effect of PTSS). *p < 0.05, **p < 0.01, and ***p < 0.001.

Delving into how these variables are related, Figure 2 shows the predictive model tested and the results obtained, and Table 5 shows the different estimates and effects with their confidence interval. Adjustment indices were optimal [χ^2^ = 83.10, *df* = 40, χ^2^/*df* = 2.08, CFI = 0.98, TLI = 0.98, RMSEA = 0.04 (90% CI = 0.03, 0.06), SRMR = 0.04]. A total of 19.37% (95% CI = 0.11, 0.34; *p* < 0.001) of the variance of PTG at T3 could be predicted by the levels of resilience, PTSS, and social participation during the general home confinement (measured in T2). The relationship between resilience and PTG was mediated by PTSS, with a statistically significant indirect effect (–0.14 [95% CI = –0.20, –0.90], *SE* = 0.03, *p* < 0.001. This mediation effect was total, suggesting that resilience influences PTG levels only *via* its effect on PTSS.

**TABLE 5:** Standardized estimates of the different paths and effects of the model.

Direct Paths					95% CI		
			Estimate	*SE*	Lower	Upper	*p*
Resilience	→	PTSS	–0.43	0.05	–0.51	–0.33	<0.001
Resilience	→	PTG	0.02	0.05	–0.08	0.13	0.07
PTSS	→	PTG	0.32	0.05	0.22	0.43	<0.001
Social Participation	→	PTG	0.31	0.09	0.12	0.49	<0.001

**Model effects**			**Estimate**	***SE***	**Lower**	**Upper**	***P***

Direct effect			0.02	0.05	–0.08	0.13	0.65
Indirect effect			–0.14	0.03	–0.20	–0.09	<0.001
Total effect			–0.11	0.05	–0.21	–0.02	0.02

## Discussion

The health crisis derived from COVID-19 has generated a substantial psychological impact, reflected in a significant prevalence of moderate levels of PTSS (Rodríguez-Rey et al., 2020) and, without detracting from the severity of these harmful consequences, positive psychological changes such as PTG have also been observed (e.g., Yeung et al., 2022). However, studies evaluating this consequence, although increasingly prevalent, continue to be scarce and mostly cross-sectional, being unable to study whether this change is maintained longitudinally, and which COVID-19-related variables are relevant long-term predictors of PTG development. In this sense, there are relevant contextual variables, such as carrying out collective activities and rituals, that had not been previously studied in the context of COVID-19. Additionally, there is notable controversy in the literature regarding how some key variables relate to PTG in the context of trauma, such as PTSS and resilience (Shakespeare-Finch and Lurie-Beck, 2014; Rzeszutek and Gruszczyńska, 2018). Thus, the objective of this work was to longitudinally explore the level of PTG generated by the COVID-19 crisis, its temporal stability, and the variables related to PTG, and to test a predictive model of the theoretical paradigm of the construct.

Our results found that a significant number of participants (around 20%) showed moderate or higher PTG as a result of the COVID-19 crisis, with no significant changes in their evolutionary trend between July and November 2020. This supported the hypothesis that the change persisted over time and, therefore, had a lower probability of it being a sporadic illusory phenomenon, as some critics have suggested (Kaur et al., 2017). However, we did not evaluate the possible existence of different trajectories in its evolution, as referred in previous studies (Cheng et al., 2020; Zhao et al., 2021) so this result should be taken with caution.

Regarding the sociodemographic profile with greater PTG, it was that of the women and younger age groups, similar to that reported in previous studies (Helgeson et al., 2006; Vishnevsky et al., 2010; Wu et al., 2019; Kalaitzaki, 2021). Furthermore, the variables significantly related to PTG were mostly consistent with previous studies. In this sense, those who experienced a more adverse situation presented greater PTG (Helgeson et al., 2006). Specifically, the variables associated with higher PTSS in T1 (Rodríguez-Rey et al., 2020) were related to higher PTG in T3: losing a job or stopping work due to COVID-19, perceived risk of, or having, a salary reduction, higher levels of concern, having a non-cohabiting partner, and being single. This was consistent with previous research (Hyun et al., 2021; Ikizer et al., 2021; Na et al., 2021; Yeung et al., 2022). Likewise, those participants who suffered the loss of a loved one or had symptoms compatible with COVID-19 presented higher levels of PTG, also in accordance with the literature (Prieto-Ursúa and Jódar, 2020; Zhou et al., 2020; Yeung et al., 2022; Zhang et al., 2022).

In addition, we were able to identify some factors related to higher PTG that did not necessarily imply greater PTSS: a greater number of hours dedicated to leisure in T2, having exercised physically during the general home confinement of T1, and greater participation in social activities in T2. These results were consistent with the literature. On the one hand, this supports previous results on the facilitating role of leisure activities, especially physical exercise, in PTG (Chun and Lee, 2010; Chen et al., 2020; Zhang et al., 2020). On the other hand, it demonstrates the positive relationship of social support and PTG (Prati and Pietrantoni, 2009; Mo et al., 2021; Wu et al., 2021). However, not all forms of social participation had the expected effect, which may be because the measurement method employed did not necessarily reflect community cohesion or perceived social support. For example, community cooperation activities were not related to PTG, maybe some participants felt obliged to cooperate without being intrinsically motivated to do so, consistently with the stages crossed after a traumatic event as a community, where initially there is a boom in participation in altruistic and solidarity activities that eventually declines (Páez et al., 2013). Future studies could specifically assess the role of the levels of experienced cohesion or perceived social support, in addition to assessing the possible component of social desirability.

Regarding the relationship between resilience and PTG, a full mediation effect by PTSS was found. According to our results, resilience was a protective factor against PTSS, and as such was related to a lower PTG. Although these findings contradict those of some previous works (Gouzman et al., 2015; Dong et al., 2017; Rzeszutek and Gruszczyńska, 2018) they support the theoretical model that maintains that those individuals with greater resilience will experience lower PTSS and, therefore, lower PTG (Westphal and Bonanno, 2007; Tedeschi and McNally, 2011). Thus, the relation between PTG and resilience appears to be complex. A possible explanation for the existence of both a direct relation between PTG and resilience in previous studies and an inverse one in ours could be that people who experienced higher PTSS and PTG in the face of adversity develop greater resilience to future crises (Tedeschi and McNally, 2011) and, therefore, future life adversities could cause them less PTSS and PTG. It would be useful to evaluate this hypothesis longitudinally in future research.

Our findings also showed that social participation had a significant effect on the development of PTG, although weak, which could be due to the aforementioned reasons. In any case, the model supports that social participation can be understood as a positive experience that, after a traumatic event, allows the reconstruction of positive beliefs, generating greater PTG (Prati and Pietrantoni, 2009; Mo et al., 2021). These results are in line with those of Northfield and Johnston (2021), showing that the effect of PTSS on PTG within the context of COVID-19 is enhanced by social support.

### Limitations of the study and future directions

Our study evaluated the development of PTG in a specific context, that of the health crisis caused by COVID-19. It is one of the few studies, to date, that addressed this issue longitudinally, which is relevant at a theoretical and practical level. However, the study is not without limitations that must be mentioned. On the one hand, despite the large number of participants, the sample did not equally represent the characteristics of the Spanish population; there was low participation of individuals over 65 years of age, which could be due to the online format of the evaluations. In addition, the participation of women was significantly higher than that of men, which has been recurrently found in previous works (Korkeila et al., 2001), as women seem to be more willing to collaborate with research. Nor can we rule out the possibility that the sample loss throughout the various evaluations was due to specific and non-random factors, limiting the generalization of the results found. Also, the measure of social participation may not adequately reflect the subjective social support perceived by the participants as it was a behavioral measure, so it would be advisable to complement it with a standardized instrument in future work.

Regarding the temporal stability of PTG, we found no differences over time in PTG, supporting that this change is not temporary and illusory as had been suggested (Kaur et al., 2017), but in future research, this measure could be complemented with actions and behaviors that could evidence such change to a greater extent. For example, in COVID-19 patients, rethinking their life priorities resulted in wanting to spend more time with their families, exercise more, lead a healthier life, etc. (Zhang et al., 2022). However, specific studies need to be carried out to identify which behaviors would be an appropriate reflection of experiencing PTG, since the manifestations could be different for each person; some people show significant growth in the religious field (Prieto-Ursúa and Jódar, 2020), while for others, this area does not seem to be relevant (Garrido-Hernansaiz et al., 2022). Additionally, it is possible that the value of growth on a personal level is intangible behaviorally, but valuable in itself.

### Practical implications

Our results have practical implications that can be considered to prevent and treat psychological distress due to the COVID-19 health crisis. In the first place, they reflect the need to adopt measures that meet the current needs of the population, since, although part of the sample reports PTG, this does not seem to cushion the negative consequences that the health crisis has had on mental health. Regarding future crises, the promotion of collective activities that could encourage community cohesion would be a measure that could facilitate the development of PTG and prevent psychopathology (Rodríguez-Rey et al., 2020). For its part, resilience acts as a protective factor against experiencing PTSS, thus adopting measures that encourage its development could be a possible preventive measure for future crises. To this end, the meta-analysis of Liu et al. (2020) found that interventions based on social support (e.g., promoting a support network) and evidence-based interventions (e.g., Cognitive Behavioral Therapy) fostered resilience building, such that, without downplaying the need for individualized attention, a community and social approach could favor the development of both PTG and resilience.

## Conclusion

This study is one of the few that longitudinally contemplated the development of PTG in the context of COVID-19, being key to understanding its development in this context. This made it possible to assess the temporal stability of PTG, supporting that it is not a temporary and illusory change. In addition, it was possible to identify contextual variables of COVID-19 related to higher levels of PTG. These variables were not only adverse (e.g., losing a loved one), but also protective (e.g., physical exercise, social participation). In addition, this study sheds some light in relation to one of the most controversial questions in this field, which refers to the mechanisms and variables related to the development of PTG (Schubert et al., 2016; Rzeszutek and Gruszczyńska, 2018). In this regard, we proposed a theory-based predictive model which supports that resilient people – those who are less likely to be severely affected by adverse events – would experience less PTG than those who suffer more due to adversity. This finding, however, is contrary to what has been found in most previous studies. Our findings should be considered in the design and stipulation of measures for future crises. It is pertinent to develop preventive psychosocial and intervention measures that can foster resilience (as a protective factor against PTSS) and the development of PTG.

## Data availability statement

The raw data supporting the conclusions of this article will be made available by the authors, without undue reservation.

## Ethics statement

The studies involving human participants were reviewed and approved by the Ethics Committee at Universidad Pontificia Comillas. The patients/participants provided their written informed consent to participate in this study.

## Author contributions

PC-C: conceptualization, methodology, formal analysis, investigation, data curation, writing – original draft preparation and review and editing, visualization, and supervision. RR-R: conceptualization, methodology, formal analysis, investigation, resources, writing – original draft preparation and review and editing, visualization, supervision, project administration, and funding acquisition. HG-H: conceptualization, methodology, investigation, writing – review and editing, visualization, and supervision. SC: conceptualization, investigation, writing – review and editing, visualization, and supervision. All authors read and agreed to the final version of the manuscript.
