# Supplementary material for: Prediction of post-traumatic growth in the face of the COVID-19 crisis based on resilience, post-traumatic stress and social participation: A longitudinal study
Source: Front Psychol. 2022 Aug 11;13:985879. doi: 10.3389/fpsyg.2022.985879 (PMC9430662; doi:10.3389/fpsyg.2022.985879)
Supplement: Supplementary file 1 [file Data_Sheet_1.docx]

Supplementary Material

Supplementary Table 1. Participants lost to follow up in T1-T2 and T2-T3

|  | Lost to follow up T1-T2 | | | | | Lost to follow up T2-T3 | | | | |
| --- | --- | --- | --- | --- | --- | --- | --- | --- | --- | --- |
|  | T1-T2  M (SD) | Only T1  M (SD) | *t^1^*/ ꭓ^2^ | *p* | *g*/V* | T2-T3  M (SD) | Only T1 and T2  M (SD) | *t^1^*/ ꭓ^2^ | *p* | *g*/V* |
| Gender^2^ |  |  | 4.55 | 0.03 | 0.04* |  |  | 1.50 | 0.22 | 0.04 |
| Male | 186 (21.8) | 558 (25.5) |  |  |  | 122 (20.7) | 64 (24.4) |  |  |  |
| Female | 666 (78.2) | 1627 (74.5) |  |  |  | 468 (79.3) | 198 (75.6) |  |  |  |
| Age | 33.83 (12.57) | 31.5 (13.04) | -4.48 | < 0.001 | -0.18 | 35.02 (12.9) | 31.46 (12.87) | -6.03 | < 0.001 | -0.28 |
| PTSS | 27.91 (19.52) | 28.04 (18.42) | -0.17^1^ | 0.87 | -0.01 | 24.76 (17.06) | 28.06 (18.49) | 2.47^1^ | 0.01 | 0.19 |
| PTG |  |  |  |  |  | 14.63 (9.29) | 16.33 (9.9) | 2.41 | 0.02 | 0.18 |

^1^ Homoscedasticity could not be assumed for these variables.

^2^ Given the low number that responded "other", only men and women were included. Chi-Squared and Cramer’s V coefficients are reported (interpretation: small < 0.02, medium < 0.06, large > 0.06).

* Cramer’s V was statistically significant *p* < .05.

Supplementary Table 2. Results of multiple linear regression predicting PTG in T3 from sociodemographic variables.

|  | B | SE | β | *t* | *p* |
| --- | --- | --- | --- | --- | --- |
| Constant | 7.30 | 1.21 |  | 6.03 | < 0.001 |
| Gender |  |  |  |  |  |
| Being male | Reference group | | | | |
| Being female | 4.20 | 0.97 | 0.18 | 4.33 | < 0.001 |
| Marital status |  |  |  |  |  |
| Married or cohabiting with a partner | Reference group | | | | |
| In a relationship but not cohabiting | 2.50 | 1.16 | 0.11 | 2.16 | 0.03 |
| Separated/ divorced | 1.67 | 2.28 | 0.03 | 0.73 | 0.46 |
| Single | 2.48 | 1.05 | 0.12 | 2.37 | 0.02 |
| Age groups |  |  |  |  |  |
| 18-24 years old | 2.32 | 1.33 | 0.11 | 1.74 | 0.08 |
| 25-34 years old | 3.07 | 1.18 | 0.15 | 2.60 | 0.01 |
| 35-44 years old | Reference group | | | | |
| 45-54 years old | 2.45 | 1.32 | 0.10 | 1.86 | 0.06 |
| 55-64 years old | 4.04 | 1.75 | 0.11 | 2.31 | 0.02 |
| 65-74 years old | 2.03 | 2.81 | 0.03 | 0.72 | 0.47 |

Note. *R*^2^ = 0.07; *F* (9, 582) = 4.71; *p* < 0.001
